# A DIVA vaccine strain lacking RpoS and the secondary messenger c-di-GMP for protection against salmonellosis in pigs

**DOI:** 10.1186/s13567-019-0730-3

**Published:** 2020-01-10

**Authors:** Carmen Gil, Cristina Latasa, Enrique García-Ona, Isidro Lázaro, Javier Labairu, Maite Echeverz, Saioa Burgui, Begoña García, Iñigo Lasa, Cristina Solano

**Affiliations:** 1 Laboratory of Microbial Pathogenesis, Navarrabiomed-Universidad Pública de Navarra (UPNA)-Complejo Hospitalario de Navarra (CHN), IdiSNA, Irunlarrea 3, 31008 Pamplona, Navarra Spain; 2 Recombina S.L., 31192 Mutilva, Navarra Spain; 3 Instituto Navarro de Tecnologías e Infraestructuras Agroalimentarias-INTIA, 31610 Villava, Navarra Spain

## Abstract

Salmonellosis is the second most common food-borne zoonosis in the European Union, with pigs being a major reservoir of this pathogen. *Salmonella* control in pig production requires multiple measures amongst which vaccination may be used to reduce subclinical carriage and shedding of prevalent serovars, such as *Salmonella enterica* serovar Typhimurium. Live attenuated vaccine strains offer advantages in terms of enhancing cell mediated immunity and allowing inoculation by the oral route. However, main failures of these vaccines are the limited cross-protection achieved against heterologous serovars and interference with serological monitoring for infection. We have recently shown that an attenuated *S.* Enteritidis strain (ΔXIII) is protective against *S.* Typhimurium in a murine infection model. ΔXIII strain harbours 13 chromosomal deletions that make it unable to produce the sigma factor RpoS and synthesize cyclic-di-GMP (c-di-GMP). In this study, our objectives were to test the protective effects of ΔXIII strain in swine and to investigate if the use of ΔXIII permits the discrimination of vaccinated from infected pigs. Results show that oral vaccination of pre-weaned piglets with ΔXIII cross-protected against a challenge with *S.* Typhimurium by reducing faecal shedding and ileocaecal lymph nodes colonization, both at the time of weaning and slaughter. Vaccinated pigs showed neither faecal shedding nor tissue persistence of the vaccine strain at weaning, ensuring the absence of ΔXIII strain by the time of slaughter. Moreover, lack of the SEN4316 protein in ΔXIII strain allowed the development of a serological test that enabled the differentiation of infected from vaccinated animals (DIVA).

## Introduction

Salmonellosis remains the second most common zoonosis in humans in the European Union (EU), with 91 662 confirmed cases in 2017. Despite national control programmes, in recent years, the declining trend of salmonellosis cases has levelled off and the number of reported cases in the EU has not shown any statistically significant decrease. Moreover, *Salmonella* is still responsible for the highest number of food-borne outbreaks in the EU, with eggs, pig meat and derived products being main sources of this pathogen [1]. The European Commission set the criteria to control *Salmonella* infections within the poultry sector, resulting in a correlated reduction in the human cases associated with the consumption of eggs [2, 3]. On the contrary, proposals concerning the monitoring and control of *Salmonella* in pigs have been dropped [4], but still, regulations in the swine sector should follow to tackle *Salmonella* infection in pigs [5]. The most common serovar at EU level causing human food-borne infections from pork is *Salmonella enterica* serovar Typhimurium (*S.* Typhimurium), being widely prevalent along the entire pig chain [1]. Therefore, it is assumed that control measures should be based on actions taken throughout the production chain, including a combination of measures aimed at preventing horizontal and vertical transmission, with the final objective of producing *Salmonella*-free animals. At the pre-harvest level, measures can be addressed to the prevention of introduction of *Salmonella* into the herd; the prevention of in-herd transmission; and the increase of resistance to infection [6, 7]. In this regard, *Salmonella* vaccines are currently regarded as an adjunct to other on-farm control measures [7–10], by helping to prevent *Salmonella* colonization and the development of a carrier state, characteristic of pigs colonized by non-adapted serovars, such as *S.* Enteritidis and *S.* Typhimurium [7, 11].

In recent years, increasing numbers of live *Salmonella* attenuated vaccines have been developed for pigs vaccination [7–9, 12–17], although most of them are not yet authorized. While this type of vaccines are claimed as the most effective means of immunoprophylaxis against *Salmonella* [11], there are major drawbacks that must be taken into account. First, vaccines are usually serovar specific, providing limited protection against infections with *Salmonella* belonging to other serovars; second, vaccination may interfere with established serological monitoring programs, making it difficult to differentiate between vaccinated and infected animals; and third, attenuated vaccine strains may reverse to virulent, unsafe forms [9].

We recently published a report detailing the analysis of an attenuated *S.* Enteritidis vaccine strain, referred to as ΔXIII, that protected mice against a lethal oral challenge of a *S.* Typhimurium virulent strain [18]. ΔXIII strain is a multiple mutant in *rpoS*, the gene encoding the master sigma factor during stationary phase and under a variety of stress conditions [19] and also in the 12 genes encoding diguanylate cyclase proteins responsible for the synthesis of the second messenger bis-(3′-5′)-cyclic dimeric GMP (c-di-GMP) [20, 21]. Absence of c-di-GMP in ΔXIII leads to a moderate attenuation [18] whilst the additional mutation in *rpoS* results in a highly attenuated strain [18, 22]. Moreover, the secondary messenger c-di-GMP is a key molecule in the transition from a planktonic to a biofilm lifestyle [23] since it is required for the synthesis of cellulose, the main exopolysaccharide of the *Salmonella* biofilm matrix [24–26]. Hence, ΔXIII strain is unable to form a biofilm and is sensitive to chlorine treatment, ultraviolet light irradiation, heavy metal stress and desiccation conditions [18, 27]. In our previous report carried out in mice, the vaccine candidate showed several qualities of a promising veterinary *Salmonella* vaccine, such as the induction of a cellular/humoral balanced, long-lasting immune response; cross protection against the non-homologous serovar *S.* Typhimurium; low environmental persistence; improbability of virulence reversal because of the complete deletion of 13 specific genes in its chromosome and also, DIVA features that allow differentiation of infected and vaccinated animals [18].

In the current study, our objectives were to assess whether *S.* Enteritidis ΔXIII is also able to cross-protect piglets from a challenge with the heterologous serovar *S.* Typhimurium and to evaluate whether this vaccine strain can be used in swine without compromising the differentiation of infected from vaccinated animals. We demonstrate that vaccination of pre-weaned piglets with ΔXIII strain conferred protection against subsequent challenge with a multi-antimicrobial resistant *S.* Typhimurium strain, by reducing *S.* Typhimurium gastrointestinal tissue colonization and faecal shedding, both at the time of weaning and slaughter. Importantly, vaccination resulted in the induction of a serological response lacking antibodies against the diguanylate cyclase SEN4316, enabling the development of an ELISA assay for the discrimination between vaccinated and infected pigs.

## Materials and methods

### Bacterial strains and culture conditions

The vaccine candidate, *S.* Enteritidis ΔXIII, is a multiple mutant, derivative of the wild type clinical isolate *S.* Enteritidis 3934 [24, 28], carrying deletions in all genes encoding GGDEF domain proteins and in *rpoS* [18, 20, 21]. *S.* Typhimurium STM610T, a multi-antimicrobial resistant strain serologically identified and isolated from the mesenteric lymph nodes of a fattening pig at slaughter, was used as the challenge strain [29]. Bacteria were grown in LB broth and on LB agar. Media was supplemented with ampicillin (Am), 100 μg/mL, to culture and isolate the challenge strain.

To prepare the vaccine culture and the challenge strain for pig administration, a 100 mL culture (LB medium) was inoculated with 100 µL of an overnight culture in LB medium and was grown with shaking at 37 °C for 20 h. The culture was kept on ice until use for a maximum of 2 h before administration. Inoculum doses were determined immediately after infection via serial dilution and subsequent plating onto LB agar.

### Ethics statement

All procedures involving animals were performed in accordance with the European regulations regarding the protection of animals used for experimental and other scientific purposes (Directive 2010/63/EU of the Europe), under the supervision of the Ethical and Animal Welfare Committee of the Public University of Navarra, Spain (approved protocol PI-008/11).

### Sample collection from sows and processing

In order to analyse the level of *Salmonella* seroprevalence in sows of the selected farm, a serological examination to evaluate the presence of *Salmonella* antibodies was conducted on five randomly selected pregnant sows. Sow blood was collected 1 week before farrowing. After coagulation, blood samples were centrifuged for 5 min at 1500 × *g* to collect serum and kept frozen (−20 °C) until serological analyses were carried out. Colostrum samples were collected from the sows on the day of farrowing. Colostrum was collected from sows immediately after the last piglet was born, when the contractions had stopped and after the expulsion of placenta. Colostrum was collected from three random teats located in the anterior, middle, and posterior part of the udder and pooled to 5 to 10 mL. The samples were centrifuged at 13 000 × *g* for 1 h and the supernatants were collected and frozen at −20 °C.

### Experimental design of the safety trial

**Figure 1:**
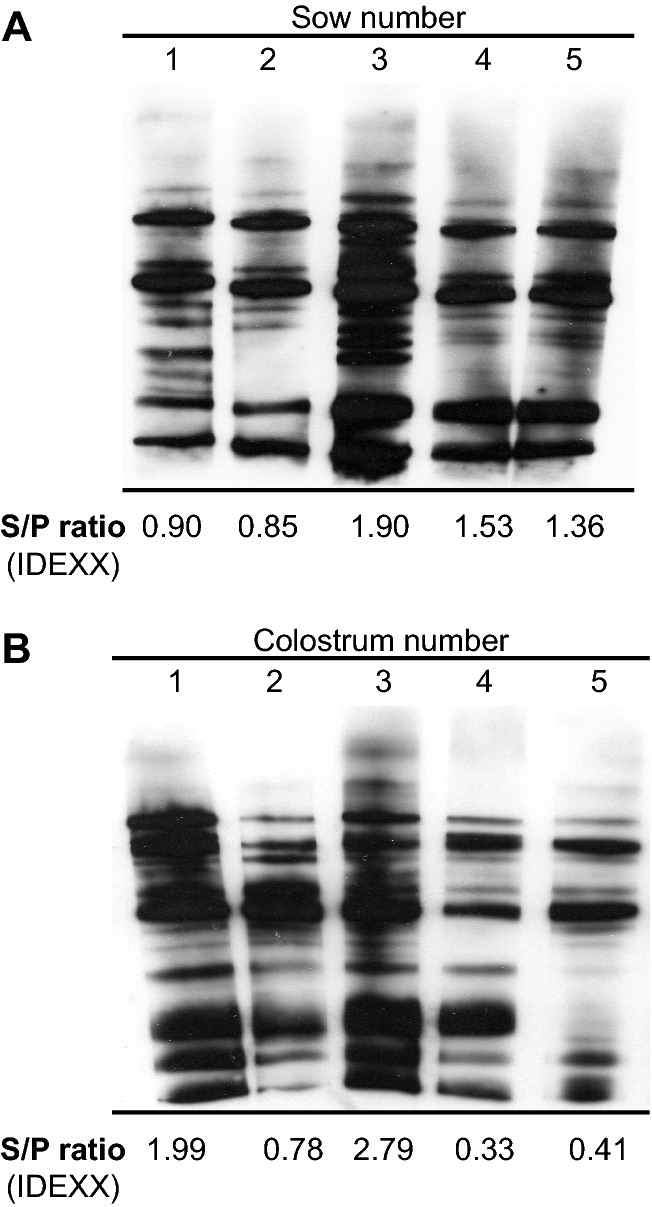
**Serological results of samples from randomly selected sows. A** Sera and **B** colostrum from five sows were analyzed by Western blot against a hot saline antigenic extract of *Salmonella* ΔXIII strain and by an LPS based IDEXX test. An S/P ratio of ≥ 0.25 is considered positive.

Three pregnant sows (Duroc) were randomly selected from a farrow-to-finish pig farm with high herd *Salmonella* seroprevalence (Fig. 1) and relocated in a newly conditioned *Salmonella* free farm, 1 week before farrowing. G Power was used to calculate the number of piglets needed to obtain statistical significance (assuming an effect size of 0.50, a power level of 0.80, and a probability level for statistical significance of 0.05). Piglets were allocated at litter level to vaccinated (total number = 21) or unvaccinated (total number = 11) groups that were housed in separate isolation units at 25 °C under natural day–night rhythm conditions.

At 4 days of age, piglets were individually checked to be *Salmonella* free by stool culture.

At 5 days of age, piglets were orally vaccinated using a bent-knob cannula and syringe to administer 1 mL of the vaccine (2.8 × 10^9^ cfu/mL) or 1 mL of sterile LB broth to vaccinated or unvaccinated groups, respectively. At 28 days of age, all piglets were weaned and vaccinated groups were orally boosted with 1.3 × 10^9^ cfu of ΔXIII strain whilst the control group received 1 mL of sterile LB broth. Piglets were euthanized at 45 days of age. Surveillance of animals was carried out along the study by monitoring the body temperature and weight to calculate the daily weight gain (DWG).

To investigate the immune response to *Salmonella* generated in vaccinated pigs, blood samples were collected at days 2, 7, 21, 35 and 42 of age and centrifuged for 5 min at 1500 × *g* to collect serum. Sera from the control group were pooled into three samples per day whilst sera from the vaccinated group were pooled into four samples per day.

### Experimental design of the vaccination and challenge trial

Four pregnant sows (Duroc) were randomly selected from the same farm described above and relocated in a newly conditioned *Salmonella* free farm, 1 week before farrowing. G Power was used to calculate the number of piglets needed to obtain statistical significance (assuming an effect size of 0.50, a power level of 0.80, and a probability level for statistical significance of 0.05). Piglets were allocated at litter level to vaccinated (total number = 24) or unvaccinated (total number = 20) groups that were housed in separate isolation units at 25 °C under natural day–night rhythm conditions. At 4 days of age, piglets were individually checked to be *Salmonella* free by stool culture.

At day 12 of age, 24 piglets from 2 litters were orally vaccinated with 2.9 × 10^9^ cfu of ΔXIII strain. 20 control piglets from 2 litters received 1 mL of sterile LB broth. At 27 days of age, all piglets were challenged with 3.4 × 10^9^ cfu of *S*. Typhimurium STM610T. Faecal samples were collected and analyzed at days 5, 13, 15, 19, 26, 28 and 32 of age. Blood samples were collected at days 5, 13, 19 and 26 of age and centrifuged for 5 min at 1500 × *g* to collect serum. Sera from the control and vaccinated group were pooled into four samples per day.

At 39 days of age, piglets were weaned, half of them were euthanized and faeces and ileocecal lymph nodes from each pig were collected. The other half remained until 180 days of age when they were slaughtered, after which faeces and ileocecal lymph nodes from each pig were collected.

### Serological analyses

Serum and colostrum IgG against *Salmonella* was detected by Western-Blot. For that, 15 µg of hot saline antigenic extract of ΔXIII strain were separated by SDS-PAGE, transferred onto Hybond-ECL nitrocellulose membranes (GE Healthcare, Buckinghamshire, UK) by electroblotting, and blocked at room temperature for 2 h with 5% skimmed milk in PBS with 0.1% Tween 20 (PBS-T) under shaking conditions. Then, membranes were exposed to immune sera or to colostrum samples, diluted 1:1000 in PBS-T containing 5% skimmed milk at 4 °C overnight. After five washes with PBS-T, membranes were incubated with rabbit anti-pig IgG HRP-conjugated secondary antibody (Thermo Fisher Scientific, Waltham, USA), diluted 1:1500 in PBS-T containing 5% skimmed milk at room temperature for 1 h and proteins were detected using Super Signal West Pico chemiluminescent substrate (Thermo Fisher Scientific).

The hot saline antigenic extract was obtained as described [30]. Briefly, live cells were suspended in physiological saline (10 g of packed cells per 100 mL) and heat was applied in flowing steam for 15 min. After centrifugation at 12 000 × *g* for 15 min, the supernatant was dialyzed for 2 days at 4 °C against several changes of deionized water. The dialyzed material was centrifuged for 5 h at 100 000 × *g*, and the pellet (hot extract) was resuspended in deionized water, lyophilized and stored at room temperature.

Also, collected sera or colostrum samples were diluted 20-fold and analyzed for *S. enterica* specific antibodies with a commercial ELISA kit based on lipopolysaccharide (LPS) O-antigens of serogroups B, C1 and D (HerdChek Swine Salmonella, IDEXX Laboratories, Hoofddorp, The Netherlands). Results are expressed as a sample to positive ratio (S:P); samples with S:P ratios ≥ 0.25 (OD% ≥ 10) were defined as positive.

### Bacteriology and strain characterization

Faeces and ileocecal lymph nodes from pigs were collected and analyzed using the standard International Organization for Standardization (ISO) 6579:2002/Amd 1:2007 method. Before the analyses, ileocaecal lymph nodes were externally decontaminated by dipping into absolute alcohol and further flaming.

Distinction between challenge and the vaccine strains was carried out by transferring all individual colonies on agar plates without antibiotics or containing ampicillin (100 μg/mL), where only the challenge strain can grow. Further confirmation of strain identification was subsequently obtained by analyzing at least five colonies from each plate by PCR, exploiting the chromosomal differences between the two strains. Oligonucleotides sen4315 (cacgattacgccaactcgagttgt) and sen4317 (gtaagataactgtgcgaag) were used in order to amplify a 632 bp fragment exclusively from ΔXIII DNA. Amplification of invA with oligonucleotides invA-fw (ggcgatattggtgtttatgg) and invA-rv (catattatcgctatcgccat) was used to amplify a 658 bp fragment from both the challenge strain and ΔXIII DNA.

### Production of recombinant SEN4316

The *sen4316* gene was amplified from *S.* Enteritidis 3934 genomic DNA with primers sen4316 BamHI-fw (ggatccatgacaacaccatcctggcg) and sen4316 SalI-rv (gtcgactcatagggcgcgcatgtcgt), using Phusion High-Fidelity DNA Polymerase (Thermo Fisher Scientific). The PCR-amplified fragment was cloned in pJET 1.2 vector (Thermo Fisher Scientific), sequenced and digested with BamHI and SalI to clone it into the pET28a vector (Novagen, Merck, Darmstadt, Germany). The resulting plasmid pET28a::*sen4316* was electroporated into *E. coli* BL21 C43 (DE3) [31]. Cultures were grown at 37 °C, 250 rpm, to an optical density (OD_600_) of 0.5, and isopropyl-d-thiogalactopyranoside (IPTG) was added to a final concentration of 0.4 mM. Cells were then grown overnight at 23 °C. Harvested cells were lysed with BugBuster HT Protein Extraction Reagent (MilliporeSigma, Burlington, USA). SEN4316 accumulated in inclusion bodies was obtained by centrifugation at 16 000 × *g* for 30 min at 4 °C and suspension of insoluble material in CTAB 1%, incubation at room temperature for 10 min with vigorous agitation and overnight incubation at 4 °C with mild agitation. The supernatant was recovered by centrifugation at 20 000 × *g* and then dialyzed against binding buffer (20 mM sodium phosphate, 500 mM NaCl, 20 mM imidazole, pH 7.4). The recombinant protein was purified with a His GraviTrap affinity column according to standard protocols (GE Healthcare). Fractions containing the protein were pooled and concentrated using Amicon Ultra-4 filter units (MilliporeSigma) (3-kDa cutoff). The concentrate was resuspended with 2.5 mL carbonate–bicarbonate buffer (pH 9.6) and the protein was further purified using a gel filtration column (PD10; GE Healthcare). Eluted protein was finally analysed by SDS-PAGE and Western-Blot and then stored in aliquots at −80 °C.

### SEN4316 based ELISA

For the SEN4316 specific based ELISA, Nunc Maxisorp 96-well plates (Thermo Fisher Scientific) were coated with SEN4316 purified protein (1 µg/well) in carbonate–bicarbonate buffer (pH 9.6) and incubated at 4 °C overnight. Plates were then washed three times with PBS containing 0.05% Tween 20 (PBS-T; pH 7.4) and blocked with 2.5% bovine serum albumin (BSA) in PBS-T at room temperature for 2 h. After three washes with PBS-T, 100 µL of sera diluted 1:100 in PBS-T containing 2.5% of BSA were added to each well and incubated at 4 °C overnight. Wells were washed three times with PBS-T and 100 µL of rabbit anti-pig IgG HRP-conjugated secondary antibody (Thermo Fisher Scientific) diluted 1:1000 were added to each well. The plates were incubated for 2 h at room temperature and then washed three times. One hundred microliters of ABTS (Sigma-Aldrich, St. Louis, USA) were added to each well and the absorbance at 420 nm was determined on an Epoch (BioTek) microplate spectrophotometer.

### Statistical analysis

Statistical analyses were performed using GraphPad Prism (version 5.01) software (GraphPad Inc., San Diego, CA, USA). A two-way analysis of variance combined with the Bonferroni test was used to analyze statistical significance in serology assays. The percentage of faeces and ileocaecal lymph nodes colonization among control and vaccinated groups was analyzed using Contingency tables for non-parametric data (Fisher’s exact test).

## Results

### Serological examination of sows

Results of both the Western blot analysis that measures the levels of IgG against a *Salmonella* ΔXIII strain antigenic extract and the IDEXX Test that determines the presence of serum antibody to *Salmonella* LPS antigen indicated that the five sows randomly selected were positive for antibodies to *Salmonella* (Figure 1A). Importantly, analysis of faecal samples obtained from 7 days before to the day of parturition indicated that none of the sows shed *Salmonella* spp. To analyze the possibility that piglets might acquire maternal immunity against *Salmonella* through the ingestion of colostrum, colostrum samples were collected from the same sows on the day of farrowing and Western blot and IDEXX analyses were performed. All colostrum samples were also positive for antibodies to *Salmonella* (Figure 1B). These results demonstrated a high seroprevalence of *Salmonella* and indicated that colostral immunity might be important in the context of piglet vaccination in the selected herd.

### ΔXIII safety and immune response in vaccinated piglets

**Figure 2:**
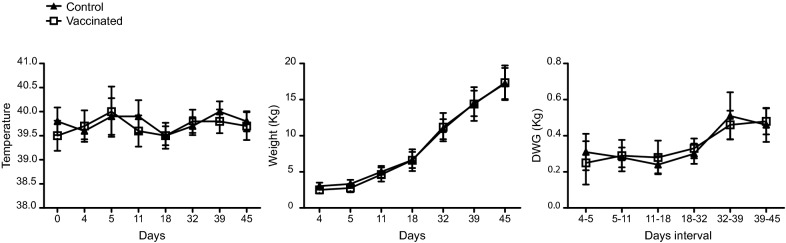
**Vaccination with ΔXIII does not have any effect in body temperature and weight gain.** Piglets from the vaccinated group (open squares) were orally vaccinated with 2.8 × 10^9^ cfu of ΔXIII at 5 days of age and boosted with 1.3 × 10^9^ cfu at 28 days of age. The control group (black triangles) received sterile LB broth. From left to right, changes in mean temperature, weight and daily weight gain (DWG) of each group are shown. No significant difference in these parameters was found between groups. Statistical analysis was carried out using a two-way analysis of variance combined with the Bonferroni test.

**Figure 3:**
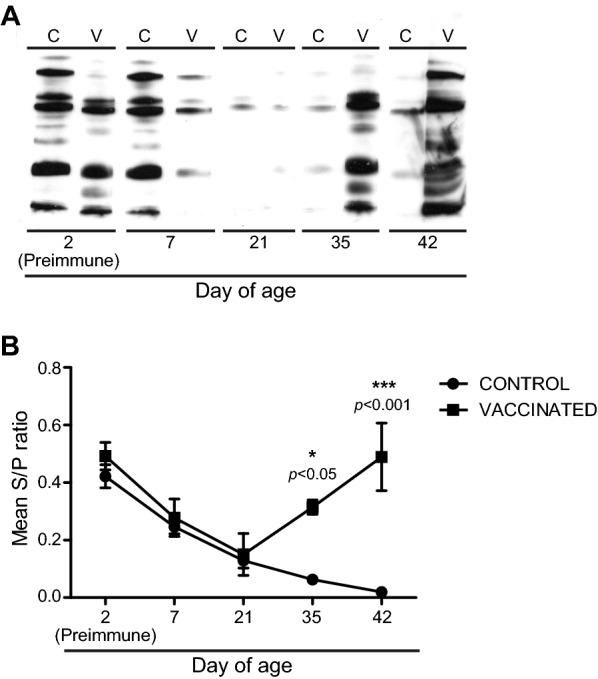
**Serological results of pigs immunized with ΔXIII strain and non-vaccinated pigs (control group).** Sera from the control group were pooled into three samples per day whilst sera from the vaccinated group were pooled into four samples per day. **A** Sera were analyzed by Western blot against a hot saline antigenic extract of *Salmonella* ΔXIII strain. In this case, an equal volume of each pool and group was mixed and the resulting samples were used for analysis. C: control; V: vaccinated. **B** Pooled sera were analyzed by an LPS based IDEXX test. An S/P ratio of ≥ 0.25 is considered positive. Statistical analysis was carried out using a two-way analysis of variance combined with the Bonferroni test. *P < 0.05; ***P < 0.001.

With the final aim of evaluating ΔXIII strain as a novel live attenuated vaccine candidate to reduce organ colonization and faecal excretion of *S. enterica* in infected pigs, we initially examined the safety of the vaccine. Results of animal surveillance showed that there were not any differences in the body temperature, weight and daily weight gain (DWG) between the vaccinated and the control groups, indicating that vaccine administration did not affect pigs health (Figure 2). Concomitantly, and in order to investigate whether ΔXIII vaccine might be able to induce a protective immune response against *Salmonella*, serum from each piglet was obtained at days 2 (preimmune), 7, 21, 35 and 42 of age and sera from each group were analyzed by Western-Blot against an antigenic extract of ΔXIII strain and by ELISA (IDEXX test) (Figure 3). The presence of antibodies at an early time in sera from both control and vaccinated pigs can be explained by the fact that the three pregnant sows used in the study were serologically positive (Figure 1) and maternal immunity was therefore acquired through the ingestion of colostrum. Maternal immunity decreased along the study and disappeared after weaning (28 days of age) in control pigs. Notably, at days 35 and 42 of age, a marked seroconversion occurred in animals immunized with ΔXIII strain. Thus, statistical analysis showed a significant difference in the antibody response against *Salmonella* LPS between pigs immunized with the ΔXIII strain and control animals.

### Vaccination and challenge trial in pigs

**Figure 4:**
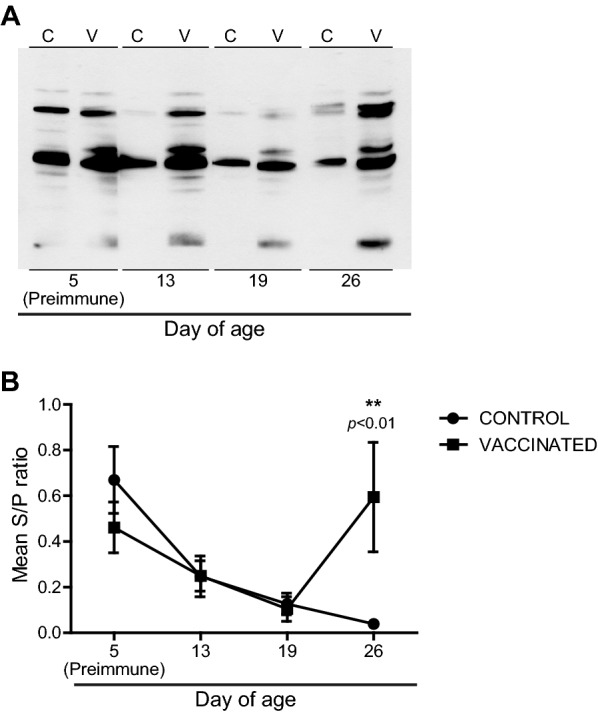
**Vaccinated piglets exhibited significant levels of serum IgG against** ***Salmonella*** **at the time of challenge.** Sera from the control and vaccinated group were pooled into four samples per day. **A** Sera were analyzed by Western blot against a hot saline antigenic extract of *Salmonella* ΔXIII strain. In this case, an equal volume of each pool and group was mixed and the resulting samples were used for analysis. C: control, V: vaccinated. **B** Pooled sera were analyzed by an LPS based IDEXX test. An S/P ratio of ≥ 0.25 is considered positive. Statistical analysis was carried out using a two-way analysis of variance combined with the Bonferroni test. **P < 0.01.

Once safety and immunogenicity of ΔXIII strain were evidenced, a vaccination trial was performed. Based on the results presented above, showing passive immunity derived from the ingestion of colostrum, and in order to optimize ΔXIII strain usefulness in vaccination field trials, piglet vaccination was delayed to day 12 of age so as to avoid interference with maternal immunity and also, vaccination was limited to a single dose. Analysis of sera obtained at days 5 (preimmune), 13, 19 and 26 of age by Western-Blot against an antigenic extract of ΔXIII strain and by ELISA (IDEXX test) (Figure 4) showed a significant seroconversion at day 26 of age in animals immunized with ΔXIII strain. Also, evaluation of *Salmonella* faecal shedding confirmed that control piglets did not acquire *Salmonella* until challenge and that vaccinated animals stopped shedding ΔXIII strain before challenge (two piglets were positive at 19 days of age; none were positive at 26 days of age). At 28 days of age, 1 day post-challenge, 45% and 62.5% of control and vaccinated pigs, respectively, were positive for faecal shedding of the challenge strain. These percentages increased to 100% and 87.5% in samples of control and vaccinated pigs, respectively, at 32 days of age.

At 39 days of age, immediately post weaning, half of the animals were sacrificed and results on *Salmonella* isolation showed that, 100% of control animals were colonized by *S.* Typhimurium STM610T. In contrast, vaccinated animals showed a faecal shedding and prevalence in ileocaecal lymph nodes of 83.3% and 66.6% respectively (Table 1). At slaughter age (180 days of age), 80% of control animals shed *Salmonella* and 40% of control ileocaecal lymph nodes were colonized. Vaccination with ΔXIII decreased *Salmonella* prevalence on both samples by half (Table 1). Although due to the sample size, colonization differences in control and vaccinated animals were non-significant when data were analyzed by the Fisher’s exact test, a trend towards significance was observed when the relative risk was calculated (Table 1). Also, sample analysis showed that none of the vaccinated pigs shed the vaccine strain or carried it in their lymph nodes at weaning and at slaughter age.

**Table 1:** **Percentage of** ***Salmonella*** **positive ileocaecal lymph nodes and faecal samples of vaccinated and control pigs**

	Post weaning		Slaughter age	
	ILN	Faeces	ILN	Faeces
	No. (%) positives^a^	No. (%) positives^a^	No. (%) positives^a^	No. (%) positives^a^
Control pigs	10/10 (100)	10/10 (100)	4/10 (40)	8/10 (80)
Vaccinated pigs	8/12 (66.6)	10/12 (83.3)	2/12 (16.6)	5/12 (41.6)
P^b^	0.096	0.480	0.347	0.099
RR^c^	1.5	1.2	2.4	1.9

^a^No. and percentage of *Salmonella* positive samples.

^b^Statistical significance (*P* values) between control vs vaccinated groups was analyzed using Contingency tables for non-parametric data (Fisher’s exact test).

^c^Relative risk.

These findings demonstrated that vaccination of piglets with ΔXIII strain reduces faecal shedding and ileocecal lymph nodes colonization, following infection with a virulent strain of *S.* Typhimurium, both at post weaning and slaughter stages.

### Evaluation of the absence of the *sen4316* gene in ΔXIII strain as a DIVA marker

**Figure 5:**
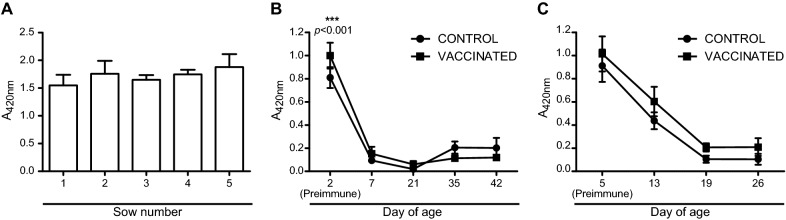
**Vaccination with ΔXIII strain elicits a DIVA humoral immune response in pigs.** SEN4316 based ELISA of sera from **A** the five randomly selected sows analysed in this study. Error bars represent standard deviation between triplicate wells; **B** control and vaccinated pigs of the present safety trial. The data represent the mean and standard deviation of duplicate measurements of every pool analysed; **C** control and vaccinated pigs of the present vaccination trial. The data represent the mean and standard deviation of duplicate measurements of every pool analysed. Statistical analysis in **B** and **C** was carried out using a two-way analysis of variance combined with the Bonferroni test.

We have previously demonstrated that ΔXIII can be considered a DIVA vaccine since the diguanylate cyclase protein SEN4316, that is absent in ΔXIII strain, enables discrimination of infected and vaccinated animals, after an oral administration of either a wild type or ΔXIII strain to BALB/c mice [18]. Specifically, antibodies directed against the SEN4316 protein were developed upon infection with a wild type strain and not upon vaccination with the ΔXIII vaccine strain [18]. To investigate the functionality of the marker SEN4316 in field vaccination trials, we analysed all sera used along this study using a specific “in-house” ELISA assay in which a 6-His-tagged recombinant version of SEN4316 was used as the bound antigen. Firstly, sera obtained from sows were probed and, as expected, and since all five sows were positive for antibodies to *Salmonella* (Figure 1A), they were also positive for antibodies directed against the SEN4316 protein (Figure 5A). Then, pooled sera from control to vaccinated animals of the safety trial (Figure 3) were examined. In this case, animals were never challenged with a wild type strain, and thus, neither control nor vaccinated pigs were serologically positive for SEN4316 at the end of the safety study (Figure 5B). Finally, sera coming from the vaccination trial (Figure 4) were tested. Taking into account that all sera corresponded to the pre-challenge stage, both control and vaccinated pigs showed titers of antibodies against SEN4316 that decreased gradually along time and never seroconverted (Figure 5C). Note that high titers shown by sera obtained at an early time (Figures 5B and C) correspond to maternal immunity acquired through the ingestion of colostrum.

## Discussion

It is nowadays widely accepted that vaccination for *Salmonella* in modern pig production can play an important role in the intervention in high prevalence herds [7–10, 32]. In a previous study, we constructed a novel live attenuated *Salmonella* Enteritidis vaccine candidate, named ΔXIII, and showed its efficacy against *Salmonella* Typhimurium in a vaccination-challenge analysis carried out in BALBc mice [18]. The results presented herein extend these findings to pigs vaccination based on ΔXIII qualities that make it a promising veterinary vaccine [33]. There are few studies examining the outcome of vaccination of pre-weaned piglets [9, 13, 34] and also, there is little information on candidate vaccines controlling *Salmonella* carriage and shedding by the time of slaughter, which is when *Salmonella* may lead to pig products contamination, resulting in human disease [9]. Thus, our study focused on vaccination of pre-weaned piglets from a farrow-to-finish pig herd with high *Salmonella* seroprevalence and extended sampling to slaughter age. As regards the study design, we focused on the oral administration of a single dose of the vaccine and also of a single high-dose challenge of a heterologous serovar, that is *S.* Typhimurium, followed by sampling to assess vaccine effects. Piglets used in our study exhibited high *Salmonella* IgG titers derived from passive immunity of the sows. In this regard, there are previous studies demonstrating that suckling pigs with higher antibody titres show an improved resistance when challenged with *Salmonella* [35–37]. On the contrary, De Ridder et al. [13] showed that amongst the herds analysed, a herd with the highest maternal *Salmonella enterica* antibody levels at vaccination was the only herd without significantly decreased *Salmonella* excretion at finishing state. Taking all this into account, we decided to delay vaccination to day 12 of age in order to avoid interference between maternal immunity and oral vaccination.

Overall, our findings provide evidence that vaccination with ΔXIII strain may be a suitable option for a *Salmonella* reduction strategy in farrow-to-finish pig herds. First, the candidate vaccine was safe and did not yield any adverse reactions in the vaccinated pigs. Second, after only one dose, ΔXIII strain generated a response capable of reducing *Salmonella* prevalence in both mesenteric lymph nodes and faeces, suggesting that a fine balance between sufficient attenuation and sufficient immune response stimulation was achieved. It is very important to note that this candidate vaccine addresses the major hurdle of cross-protecting against heterologous serovars, by providing protection against the most prevalent serovar in pigs, *S.* Typhimurium. Further trials are needed to evaluate cross-protection against other serovars such as the swine pathogen *S.* Cholerasuis [38]. Third, reversion to the wild-type phenotype is extremely unlikely in ΔXIII strain due to the complete deletion of 13 genes. Notably, these 13 deletions were carried out through a strategy that guarantees the lack of any trace of exogenous DNA [20, 21] and therefore, ΔXIII strain cannot be classified as a genetically modified organism. Fourth, vaccinated animals stopped shedding ΔXIII strain 2 weeks after vaccination, and at weaning and slaughter age, none carried ΔXIII in the lymph nodes. Moreover, ΔXIII strain is sensitive to environmental hazards [18, 27], facilitating its elimination from the farm environment when excretion by vaccinated animals occurs.

Since most of the control programs include surveillance of the herd status by monitoring *Salmonella* serological status in finishing pigs at market weight [7], a very important additional aspect of ΔXIII is that vaccination with this strain induces a response that is distinguishable from that produced by a natural *Salmonella* infection. Few *Salmonella* DIVA vaccines for pigs vaccination have been constructed [17, 38, 39]. The rationale behind this type of vaccines is that lack of specific antigens or epitopes allows the use of a serological test to discriminate infected from vaccinated animals. In the case of ΔXIII strain, we already proposed the SEN4316 diguanylate cyclase as a negative selectable marker because it allows the serological discrimination of vaccinated from infected mice and also because SEN4316 is conserved in all *S. enterica* serovars and absent in *E. coli* and other gram negative bacteria [18]. The present study confirms the use of a SEN4316 based ELISA to differentiate infected from ΔXIII vaccinated pigs.

Overall, our results indicate the efficacy of ΔXIII strain as a candidate mucosal DIVA vaccine against salmonellosis in pigs. Nevertheless, a *Salmonella* ΔXIII strain vaccination trial involving natural challenge with a large number of pigs is needed to assess vaccination relevance in field conditions. Also, future work might be conducted to assess ΔXIII strain use as a carrier for recombinant antigens in order to exploit its potential as a mucosal multivalent vaccine candidate [9].

## Data Availability

All data generated or analysed during this study are included in this published article.
